# Huremović D, editor. Psychiatry of Pandemics: a Mental Health Response to Infection Outbreak: Springer International Publishing 2019; 185 pages; ISBN978-3-030-15346-5 (e-book), ISBN978-3-030-15345-8 (softcover)

**DOI:** 10.3325/cmj.2020.61.306

**Published:** 2020-06

**Authors:** Dora Polšek

This book, dealing with a specific and so far underdeveloped field of psychiatry, is intended for a general audience interested in the overall mental health implications of a pandemic. Although parts of the text can be understood by non-medical readers, the sections focusing on medication and specific symptoms could be harder to follow without a medical background.

The series of chapters was envisaged as an all-encompassing review of the available research in the narrow niche of psychiatric consequences of a pandemic. Serendipitously, the book was published before the outbreak of COVID-19, and at several places forebodes the emergence of a Disease X, explaining the reasons that increase the odds of this scenario. Today, several months later, it sounds as a feeble voice of a prophet, drowned by the noise of the actual coronavirus pandemic.

The book starts off by giving a brief historical background of the most widely known pandemics, so as to underline the limited information that has been available to science today. Several key reasons for knowing so little about mental health consequences of witnessing and surviving a pandemic are listed. For example, after the Spanish Flu the shock and horror of losing 50-100 million lives in a year had subsided so unusually quickly that the pandemic was dubbed the “forgotten pandemic.” This and other observations provide food for thought amid the information overload related to the current global situation.

The text goes on to cover cultural, sociological, technological, legal, and managerial aspects of a pandemic, including surprising topics such as the analysis of the psychology behind the zombie apocalypse fantasy, as well as a point-by-point guideline for treating health care staff working with infectious patients. The authors give considerable attention to the impact of social distancing and quarantine on mental health, citing research papers showing a higher incidence of depression, posttraumatic stress disorder, and other anxiety disorders in patients admitted to intensive care units, family members of infected patients, and health care workers. Many explanations presented in the book might seem intuitive and already known to the reader. However, the book’s value lies in diligent and thorough fact-checking, as well as patient and unbiased data presentation, that stand in sharp contrast with shallow and sensationalistic style of most of the articles we are served daily in our news feeds. Several chapters give an overview of psychiatric treatments and presentations of well-known illnesses. Although in line with the all-encompassing goal of this book, the information given is too simplistic for a clinical expert but too complex for a non-specialized audience, and seems out of place.

The limitation of the new and exciting field described in this book is the specificity of every pandemic when it comes to the affected population, pathology, incubation period, infectivity, mortality, and cultural context, so the conclusions drawn can be only sign posts for new solutions in an actual pandemic. However, this book provides a good review of different approaches necessary to deal with psychologic issues arising amid and in the aftermath of a pandemic and could be a compelling read for the interested audience.

Related reading: Morganstein JC, Fullerton CS, Ursano RJ, Donato D, Holloway HC. Pandemics: Health Care Emergencies, In: Ursano RJ, Fullerton CS, Weisaeth L, Raphael L, eds. Textbook of Disaster Psychiatry. Cambridge University Press: 2017; pp. 270-284.

